# Pollination mechanism in *Serapias* with no pollinaria reconfiguration

**DOI:** 10.1093/aobpla/plad054

**Published:** 2023-08-16

**Authors:** Micaela Lanzino, Anna Maria Palermo, Giuseppe Pellegrino

**Affiliations:** Department of Biology, Ecology and Earth Sciences, University of Calabria, 87036 Rende, Cosenza, Italy; Department of Biology, Ecology and Earth Sciences, University of Calabria, 87036 Rende, Cosenza, Italy; Department of Biology, Ecology and Earth Sciences, University of Calabria, 87036 Rende, Cosenza, Italy

**Keywords:** Breeding system, orchids, pollinaria reconfiguration, pollination, reproductive biology, Serapias

## Abstract

Orchidaceae, one of the most numerous families in the world’s flora, have evolved various pollination strategies to favour cross-pollination, such as deceptive pollination and pollinarium reconfiguration. Among the terrestrial orchids of the Mediterranean, only species belonging to the genus *Serapias* show a strategy defined as shelter imitation. The floral elements form a tubular structure that insects use during their resting phases. The purpose of this article was to clarify the mechanisms that guarantee pollination with particular attention to the morphological interactions between orchids and pollinators and whether pollinaria reconfiguration is necessary in the promotion of cross-pollination in *Serapias*. Breeding system experiments and hand-pollination treatments indicated that *Serapias* was highly self-compatible, shows low value of natural fruit set and is pollinator limited. Time-lapse photos showed that the pollinarium had no refolding of the stipe or caudicle after its removal from the flower. The morphology of the flower determined the attack of the pollinarium on the occiput/vertex of insect. When the insect left the flower, the pollinarium was unable to encounter the stigma. When the insect made a second visit to another flower, the pollen masses of the first pollinarium ended up on the stigma and at the same time, the insect picked up a second pollinarium. Our observations and analyses suggested that morphological interactions between flower and pollinator are crucial to the success of pollination and to prevent self-pollination and thus that pollinarium reconfiguration is unnecessary in shelter deceptive orchids, such as *Serapias* species, for the promotion of cross-pollination. *Serapias* represent a case of interactions between plant and pollinator; the formation of the tubular shape of the flower is an essential preadaptation for the development of resting site mimicry originating exclusively in *Serapias* among Mediterranean orchids.

## Teaser

Orchidaceae, one of the most numerous families in the world’s flora, have evolved various pollination strategies to favour cross-pollination. *Serapias* show a strategy defined as shelter imitation. The floral elements form a tubular structure that insects use during their resting phases. Breeding system experiments and hand-pollination treatments indicated that *Serapias* was highly self-compatible, shows low value of natural fruit set and is pollinator limited. Time-lapse photos showed that the pollinarium had no refolding of the stipe or caudicle after its removal from the flower. Results suggested that morphological interactions between flower and pollinator are crucial to the success of pollination and to prevent self-pollination and thus that pollinarium reconfiguration is unnecessary in shelter deceptive orchids for the promotion of cross-pollination.

## Introduction

Orchidaceae is the second-largest family in the plant kingdom with about 20 000 species (Dressler 1993), outnumbered only by the Asteraceae (Bremer 1994). Most orchids are native to tropical or sub-tropical areas of Asia, Central America and South America; only 15 % of them grow spontaneously in European and Mediterranean areas. Tropical species often have fleshy or fine aerial roots, which represent modifications and adaptations to epiphytic life, while European and Mediterranean orchids are terrestrial species with underground root system, made up of rhizotubers or bulbs.

Apart from some orchids showing spontaneous autogamy ability (Claessens and Kleynen 2011; Talalaj *et al*. 2017), most orchids require pollinators for fruit set. Self-pollination has many disadvantages, such as genetic defects, lack of variation and the inability of the plant to adapt to climate change. Thus, orchids have evolved two intriguing mechanisms that promote cross-pollination: deceptive pollination and pollinarium reconfiguration.

Reward reinforces associations in animals using signals, with rewards promoting sequential visits to inflorescence and increasing the possibility of self-pollination. Conversely, deceptive plants attract insects but do not reward them, thus exploiting the innate or learned associations of pollinators. In this way, rewardlessness reduces self-pollination and increases pollen export and outcrossing.

Orchids show different pollination strategies and a high number of unrewarding species compared to other angiosperms; approximately 6500 species out of 7500 deceptive species belong to Orchidaceae (Shrestha *et al.* 2020). More than 80 % (65 out of 112) of orchid genera show generalized food deception (38) or food-deceptive floral mimicry (9) and sexual deception (18) (Jersáková *et al.* 2006). To attract pollinators, food-deceptive orchids display general floral signals typical of rewarding plant species (Schiestl 2005), grow near co-flowering nectariferous species (Johnson *et al.* 2003; Pellegrino *et al.* 2008) or exhibit floral colour polymorphism (Pellegrino *et al.* 2005*a*; Kellenberger *et al.* 2019). Flowers of sexually deceptive orchids, which mimic the mating signals of female insects, attract male insects that attempt to copulate with the labellum of the orchid. Therefore, the pollinaria remain attached to the insect and are transferred to other flowers of the same species during subsequent visits.

Only one terrestrial orchid genus exhibits a particular pollination strategy called shelter imitation. In this strategy, flowers offer insects a floral tube in which to restore sleep, as a hide during windy and rainy weather (Gumprecht 1977) or for thermoregulation (Dafni *et al.* 1981; Felicioli *et al.* 1998). This mechanism appears in the Mediterranean genus *Serapias*, whose extremely dark red flowers seem to mimic the entrances of bees’ nests. The debate is still open on whether it considers this strategy deceptive or whether the shelter can represent a reward for the insect and therefore can be considered another example of the rewarding strategy of terrestrial orchids (Vereecken *et al.* 2010; Pellegrino *et al.* 2017).

In addition to pollination strategies, orchids exhibit another mechanism to promote cross-pollination called pollinarium reconfiguration (Darwin 1862). Pollinarium reconfiguration refers to the change in orientation that a pollinarium undergoes after its removal and prior to its deposition in the stigmatic area. The main type of pollinaria reconfiguration identified in Mediterranean orchids is the bending or twisting of an accessory structure (such as a stipe or caudicle) that connects the pollinium to a sticky pad (the viscidium) (Catling and Catling 1991; Peter and Johnson 2006). This folding occurs after pollinaria removal by the pollinators and before they are inserted into the stigma. Previous studies showed that the average reconfiguration time of an orchid pollinaria is positively correlated to the average time that pollinators spend visiting a single inflorescence (Peter and Johnson 2014; Lanzino *et al*. 2022) and is always greater than the time that the insect spends on a single flower, or visiting flowers of the same plant (Peter and Johnson 2009). Therefore, bending increases the possibility of cross-pollination by limiting the number of self-pollinations (autogamy and geitonogamy).

In this study, our attention is focused on the pollination biology of *Serapias*. The structure of the tube-shaped flower of *Serapias* made it difficult to directly observe the mechanism by which the pollinaria remained attached to the insect’s body during its visit to the flower. Using *Serapias lingua* and *S. vomeracea* as models, the objectives of this study were to answer the following questions: what is the mechanism that ensures the transfer of pollinaria? What is the morphological interaction between orchid flower and pollinators? Do *Serapias* pollinaria exhibit folding typical of other orchid genera? Does the pollination biology of *Serapias*, in terms of the pollinaria transfer mechanism and pollinaria bending, promote cross-pollination?

To answer these questions, the breeding system, reproductive success and pollinarium reconfiguration in natural populations of *S. lingua* and *S. vomeracea* were evaluated. Furthermore, to test the efficiency of pollination in relation to the correspondence between the pollinator and the sexual organs of the orchid, we built models of the main pollinator of *Serapias* that simulate insect visit to the flower.

## Materials and Methods

### Plant material

**Figure 1.**
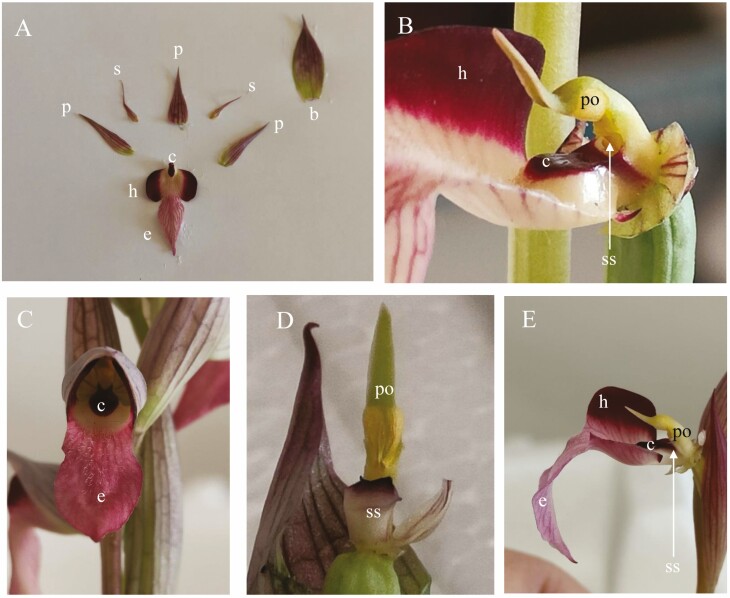
Photographs of: (A) partitioned flower of *Serapias vomeracea*; (B) gymnostemium of *S. lingua*; (C) whole flower of *S. lingua*; (D) gymnostemium of *S. vomeracea* and (E) gymnostemium of *S. lingua.* h = hypochile, e = epichile, p = petal, s = sepal, b = bractea, c = callus, po = pollinaria, ss = stigmatic surface.

*Serapias* is distributed throughout the Mediterranean region (Pridgeon *et al.* 2001) and contains about 30 species (Delforge 2006), which are characterized by a common floral morphology (Fig. 1A and C): the lateral petals and hypochile (the proximal part of the labellum) form a cap (tubular corolla). The ventral side of the basal part of the hypochile bears a callus or two distinct ridges. The epichile (the distal part of the labellum) generally slopes downward and is often pubescent (Barone Lumaga *et al*. 2012). The gynostemium, or column, is a structure resulting from the fusion of the androecium and the gynoecium (Fig. 1D). *Serapias* flowers do not show spur or nectar, have a pollinarium with pollen packed into two pollen masses, and produce olfactory signals (Pellegrino *et al.* 2012).

*Serapias lingua* and *S. vomeracea* were chosen because they belong to two different phylogenetic groups (Bellusci *et al.* 2008; Inda *et al.* 2012) and because *S. lingua* seems to have evolved a different pollination strategy that foresees the sexual attraction of pollinators (Vereecken *et al.* 2010; Pellegrino *et al.* 2017). Their tubular flower differs in morphology at the base of the hypochile, showing two guiding swellings (*S. vomeracea*) and a unique callus (*S. lingua*). *Serapias lingua* and *S. vomeracea* are widespread species, growing in arid meadows, abandoned agricultural lands and garigues. The main pollinators of both species are hymenopteran insects of four genera (*Ceratina*, *Eucera*, *Osmia* and *Tetralonia*) of the superfamily Apoidae (Dafni *et al.* 1981; Felicioli *et al*. 1998; Pellegrino *et al.* 2005*b*), as well as beetles of Oedemeridae and Lymexylidae (Pellegrino *et al.* 2005b).

### Study site

Field experiments were conducted in April–June 2022 in three sympatric populations of *S. vomeracea* and *S. lingua* growing on abandoned limestone farmland in Calabria (Southern Italy): Piano Lago (P1, elevation 640 m, 39°13ʹN, 16°17ʹE), S. Donato di Ninea (P2, elevation 700 m, 39°42ʹN, 16°02ʹE) and Arcavacata (P3, elevation 220 m, 39°20ʹN, 16°13ʹE). To minimize the effects of soil and vegetation types on our measurements, we chose sites with matched vegetation types. All populations were characterized by calcareous dry grassland (Festuco-Brometalia); *Spartium junceum*, *Cytisus sessilifolius* and *Cistus incanus* were the most common shrubs and *Festuca circummediterranea*, *Bromus erectus* and *Dactylis glomerata* were the dominant herbs. Two sites (Piano Lago and Arcavacata) occurred in a highly anthropized landscape context enclosed by high-traffic roads and their crosses and were built in the late 1960s, while the third site (S. Donato di Ninea) was moderately anthropized.

### Breeding system and reproductive success

Pollination treatments designed to investigate the breeding systems of *S. vomeracea* and *S. lingua* were performed in 2022 in P1 population. Approximately 150 unopened flowers from 30–40 plants (3–5 flowers per plant) for both species were bagged with a fine mesh cloth to exclude pollinators and then hand-pollinated. Treatments included artificial self-pollination (pollinia were taken from the same flower) (induced autogamy) or from a different flower on the same plant (induced geitonogamy), spontaneous autogamy (flower covered with a fine-meshed cloth but no manipulation) and cross-pollination (pollinia from a flower to the stigma of another flower located over 20 m away). In June, the number of capsules was counted and the ratio between the number of flowers treated/fruit produced for each hand-pollination treatment was determined.

To test the natural reproductive success, in five square grids for each populations the number of flowers that produced fruit was counted and the fruit set was determined as the mean of the ratios (number of fruits produced/numbers of flowers available) on the plants examined. To ascertain the presence of viable embryos, at least 1000 seeds from each fruit were removed from the centre of the capsule and observed under an optical microscope (×100). Seeds were assigned to two categories (viable and unviable seeds) due to the presence or absence of viable embryos. Seed set [(the number of filled seeds in sampled fruits/the number of observed seeds) × 100] was calculated for each fruit.

An ANOVA was used to partition the variation in reproductive success among populations and among plants within populations. All analyses were conducted using SPSS 16.0 for Windows. The data were analysed using descriptive statistics.

### Pollinarium reconfiguration

To test for spontaneous pollinaria reconfiguration, the pollinaria from 10 flowers from populations of each of the orchid species tested were carefully removed with toothpicks, and photographs were taken every 5–10 s until further folding movements were observed.

### Morphological interaction between flower and pollinator

To test the interaction between flower and pollinator that takes place inside the tubular structure of *Serapias* flowers, we built a small artificial carpenter bee (*Ceratina cucurbitina*), the main pollinator of *Serapias*, respecting the average size of the insect, 10 mm long and 3 mm wide (Mikát *et al.* 2022) using silver paper. To visualize the contact point between the insect head and the column, the floral elements placed on one side of the flower were cut out (Fig. 1B and E). Nine flowers for each species were used in this test. We simulated the entry of the pollinator into the flower and the contact of the head with the pollinaria and/or stigmatic cavity. Then, we moved the model to simulate the insect exiting the flower. Each simulation was photographed.

## Results

### Breeding system and reproductive success

Pollination treatments were performed in the P1 population. No flower bagged for the exclusion of pollinators developed into fruit, ruling out spontaneous autogamy for both orchid species. Artificial autogamy produced a fruit set of 88.2 and 92.5 % in *S. vomeracea* and *S. lingua*, respectively, and induced geitonogamy produced a fruit set of 84.7 and 93.6 % in *S. vomeracea* and *S. lingua*, respectively.

Cross-pollination gave a fruit set of 90 and 95 % in *S. vomeracea* and *S. lingua*, respectively. Unmanipulated plants showed low fruit set values and the populations of both species did not differ significantly in fruit production rate or viable seed percentage. In particular, the natural fruit set ranged from 18.10 to 22.40 % in *S. vomeracea* and from 19.50 to 23.20 % in *S. lingua*, while the percentage of viable seeds varied from 81.45 (±2.15) to 86.40 (±3.85) in *S. vomeracea* and from 82.25 (±2.25) to 84.85 (±2.95) in *S. lingua* (Table 1).

**Table 1.** Natural reproductive success: fruit production rate and percentage of viable seeds in three populations of *Serapias vomeracea* and *S. lingua*.

Species	Population	*N*	Fruit set (%)	Viable seeds (%)
*S. vomeracea*	Piano Lago (P1)	150	18.10	86.40 (±3.85)
	S. Donato di Ninea (P2)	138	21.80	81.45 (±2.15)
	Arcavacata (P3)	145	22.40	83.30 (±2.80)
*S. lingua*	Piano Lago (P1)	102	19.50	82.25 (±2.25)
	S. Donato di Ninea (P2)	98	23.20	84.85 (±2.95)
	Arcavacata (P3)	108	22.60	84.15 (±2.85)

### Pollinarium reconfiguration

**Figure 2.**
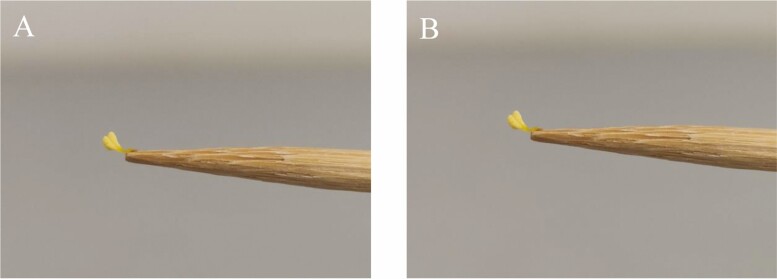
Absence of pollinaria reconfiguration in *Serapias vomeracea*. Photographs of: (A) pollinia just picked from the flower with toothpicks and (B) pollinaria after 15 min.

The time-lapse photography results indicated that all observed pollinaria of both *Serapias* species remained in the same position after 15 min, suggesting that they showed no reconfiguration (Fig. 2).

### Morphological interactions between flowers and pollinators

**Figure 3.**
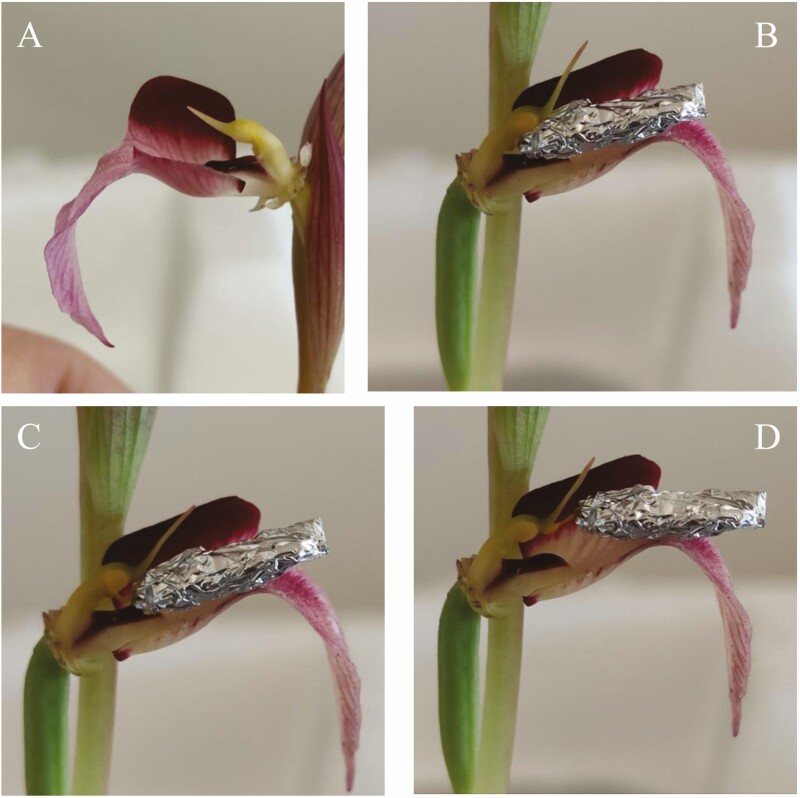
Photographs of: (A) flower of *Serapias vomeracea* without floral elements placed on one side; (B) arrival of the insect in the flower; (C) the insect drags out the pollinaria and (D) the insect leaves the flower.

**Figure 4.**
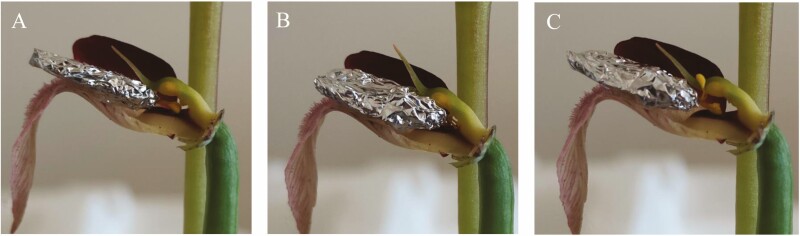
Photographs of: (A) insect visit a second flower of *Serapias vomeracea*; (B) pollinaria of the first visit touch the stigmatic cavity while a new pollinarium remain attached to the head of the insect and (C) the insect leaves the flower with two pollinaria.

When the model entered the tubular structure of the *Serapias* flower (Fig. 3A), the occiput/vertex of the insect’s head encountered the viscidium, the sticky part on the caudicle of the pollinium. The viscidium remained adhered to the insect’s head (Fig. 3B). To exit the flower, crawling backwards, the insect dragged out the pollinaria (Fig. 3C), which never managed to come into contact with the stigmatic surface of the orchid (Fig. 3D). When the insect made a second visit, bringing with it the pollinaria taken from the previous visit (Fig. 4A) the pollen masses of the first pollinaria met the stigmatic cavity, leaving pollen grains on the stigma due to their characteristic stickiness, and at the same time, the sticky part of the pollinarium of this second flower remained attached to the insect’s head (Fig. 4B). At this point, the insect left the flower, dragging both pollinaria, but those of the second flower once again had no chance of ending up on the stigma (Fig. 4C).

## Discussion

The two *Serapias* species examined showed a similar trend in reproductive success values. Fruit set differed greatly between spontaneous, induced and natural pollination (df = 3; deviance = 242.99; *P* < 0.0001). In detail, they showed a low value of natural fruit set and no population showed a percentage of reproductive success above 25 %. These data agree with those reported in other articles on the genus *Serapias* (Pellegrino *et al.* 2005; Bellusci *et al.* 2010) and with those reported for populations of unrewarding orchid species. This confirms the hypothesis that rewarding orchids are more successful in reproductive success than nectarless orchids (Neiland and Wilcock 1998). Low mean reproductive success rates have been reported for sexually deceptive orchids (Vandewoestijne *et al.* 2009; Borràs and Cursach 2021), food-deceptive orchids (Dormont *et al*. 2010; Pellegrino *et al.* 2010; Claessens and Kleynen 2011; Capó *et al.* 2021) or colour dimorphic orchids (Pellegrino *et al.* 2008; Sonne and Hauser 2014; Jersáková *et al.* 2015). High fruit set values (above 50 up to 80 %) have been reported in natural populations of rewarding orchids (Meekers and Honnay 2011; Joffard *et al.* 2020; Zhang and Gao 2021). This low level of fruit set is due to the very low pollinator visitation rates that deceptive orchid inflorescences usually experience compared to rewarding orchids (Harder and Aizen 2010).

In contrast to our results, however, relatively high fruit production has been reported in *Serapias* species; populations of *S. cordigera* in Portugal and populations of *S. vomeracea* in Israel showed fruit sets of about 50 % (Dafni *et al.* 1981; Neiland and Wilcock 1998). Differences in fruit set between such distant and different geographical areas probably reflect differences in the local composition of the entomofauna, or in anthropogenic interference habitats where the behaviours and abundance of pollinators influence the fruit set of orchid species (Pellegrino *et al.* 2015). Our examined populations are probably located in a more anthropized context, which can have negative effects on temporal fluctuations, reproductive success and population dynamics (Pellegrino and Bellusci 2014). Many Euro-Mediterranean orchids show a reduction in population size and range on regional and local scales due to habitat fragmentation by human activities (Vogt-Schilb *et al.* 2015; Geppert *et al.* 2020). Habitat fragmentations, as well as other factors such as pesticide use and climate change lead to a decline in insect diversity and biomass and a change in insect behaviour (Bergerot *et al.* 2012; Dirzo *et al.* 2014; Owens *et al.* 2020) resulting in strong pollinator limitation and plant fitness reduction (Jachuła *et al.* 2019).

The low reproductive success observed in orchids is limited by pollinators rather than resources, as demonstrated by experiments on breeding systems. First, the high fruit set achieved by artificial autogamy and artificial geitonogamy demonstrated that *Serapias* species are highly self-compatible, but the absence of spontaneous autogamy for both orchid species highlighted the need for insect visits to allow fertilization. Furthermore, the high fruit set rate after artificial cross-pollination suggests that the pollination success of the examined taxa is limited by the availability of pollinators, as reported in another deceptive orchid species.

Our experiments confirm that orchids have evolved sophisticated pollination systems and that the morphological interactions between orchids and their pollinators are crucial for pollination success (Solís-Montero and Vallejo-Marín 2017; Scaccabarozzi *et al.* 2020). The deceptive pollination strategy is essential to promote cross-pollination in orchids (Jersáková *et al.* 2006), but it does not eliminate the possibility of self-pollination. Thus, pollinarium reconfiguration is not only important for cross-pollination but is crucial in the prevention of self-pollination. Pollinarium reconfiguration is well established in all Mediterranean orchids and the time taken for pollinarium reorientation itself varies widely in different species from a few seconds (20–30 s) to several minutes (Johnson and Edwards 2000; Peter and Johnson 2006; Lanzino *et al*. 2022). Mediterranean orchids show a relationship between the time pollinators spend on inflorescence and the time for a complete bending movement. The longer the insect spends on the flower or inflorescence, the longer the folding time after withdrawal (Ostrowiecka *et al.* 2019). To the best of our knowledge, it appears that the only Mediterranean orchids genus that does not exhibit pollinarium reconfiguration is *Serapias*. Pollinarium reconfiguration completely disappears in some deceptive epiphytic orchids in tropical regions such as *Bulbophyllum*, *Broughtonia*, *Eria* and *Doritis* (Shangguan *et al.* 2008; Brodmann *et al.* 2009; Xiao-Hua *et al.* 2012). These genera show different mechanisms that prevent self-pollination, for example, the temporal variation in pollinarium size after its removal in *Bulbophyllum* species (Borba and Semir 1999).

In the case of *Serapias*, the absence of bending is due to the behaviour of pollinating insects. It has been shown that the tubular structure of the *Serapias* flower offers a refuge and shelter to insects, which, in most cases, remain inside the flower for a long time. The precise site of pollinarium placement on the pollinator is determined by several factors, such as pollinator body morphology, pollinator behaviour and flower morphology. The callus at the base of the labellum invites the insect to position itself deep in the flower and allows contact of the insect’s head with the viscidium. When the insect decides to move away from the flower, either after a few seconds or many hours, it drags the pollinarium with it, which will never encounter the stigma. Instead, when the insect visits a second flower, the pollinarium, which is directed forward on the insect’s head, perfectly insinuates itself into the stigmatic cavity thus allowing fertilization. It therefore seems that the morphology of the flower and the behaviour of the insect prevent self-pollination effectively making reconfiguration useless.

This phenomenon and the absence of bending in the populations studied cannot completely rule out the possibility of geitonogamy in *Serapias*. In fact, autogamy is impossible (or highly improbable) due to the behaviour of the insect on a single flower; there could be cases of geitonogamy if the insect, once leaving the first flower, made the next visit to a flower of the same inflorescence. Personal observations have brought to light that, after the insect has remained in the flower throughout the night or in a moment of need for rest during the day, it moves away from the inflorescence. It is likely that insects with only one visit have already received the shelter reward and do not need to visit a second flower immediately afterwards, as happens in other deceptive or rewarding orchids. Furthermore, in deceptive food orchids, for example, insects that visit one flower and receive no reward, given the absence of floral nectar, are driven to visit a second flower, a third and so on in the same inflorescence or of another plant in a fairly rapid time. In the case of nectariferous orchids, insect that receives a reward is driven to visit another flower similar to the previous one to collect more nectar to satisfy its energy needs. This is why these species, thanks to pollinarium reconfiguration and the correlation between reconfiguration time and pollinator visiting time, limit self-pollination. Insects visiting a *Serapias* flower are not driven to visit a second flower as soon as they leave the first one but will do so after many hours, in most cases after 24 h. After a long time, it is highly unlikely that the insect will choose the same flower or flower of the previously visited inflorescence with thousands of similar usable flowers. It is obvious that it cannot be excluded that the insect may return randomly to the same inflorescence, thus allowing self-pollination. Therefore, it can be concluded that the pollination biology of *Serapias*, the perfect morphological interaction between the reproductive structures of the flower and the pollinating insect, ensures cross-pollination at the expense of self-pollination without the need for pollinarium reconfiguration.

Pollinator adaptation is generally considered the main reason for floral diversification in plants. *Serapias* may represent a case of macroevolution enforced by pollinators. Food deception has been highlighted as the ancestral trait in the mostly Mediterranean orchids, and *Ophrys* and *Serapias* diversified more recently (~4.6–6.7 Mya) than other Orchidinae (~12.8–16.5 Mya) (Inda *et al.* 2012). While in *Ophrys* the production of high levels of *n*-alkenes represents the key innovation of the pollination strategy, the formation of the tubular shape of the *Serapias* flower is considered an essential preadaptation for the development of resting site mimicry originating almost exclusively in *Serapias*. The evolution of floral characteristics, especially traits involved in pollinator attraction, is under pollinator-mediated selection in many plant species; thus, flower colour and shape have been shown to have an impact on pollinator attraction (Reverté *et al.* 2016; Trunschke *et al*. 2021). Experimental modifications of the *Serapias* perianth (Pellegrino *et al.* 2017) highlighted that, even for an orchid with a refuge strategy, visual cues play a key role in the attraction of pollinators, as already demonstrated in rewarding and deceptive orchids (Jersáková *et al*. 2012; Rakosy *et al.* 2012; Sun *et al.* 2015).

## Data Availability

The data that support the findings of this study are incorporated into the article. Further inquiries can be directed to the corresponding author.

## Notes

**Natural History and Conservation. Chief Editor:** F. Xavier Picó
